# *Toxoplasma gondii* Monitoring in Liver Transplantation Patients: A Single Center Cross-Sectional Study in an Italian Hospital

**DOI:** 10.3390/pathogens9050354

**Published:** 2020-05-08

**Authors:** Barbara Pinto, Federica Lotti, Stefania Petruccelli, Paola Carrai, Paolo De Simone, Fabrizio Bruschi

**Affiliations:** 1 Department of Translational Research and New Technologies in Medicine and Surgery, University of Pisa, 56126 Pisa, Italy; barbara.pinto@dps.unipi.it (B.P.); fedina.lotti@gmail.com (F.L.); 2 Hepatobiliary Surgery and Liver Transplantation Unit, University of Pisa Medical School Hospital, 56124 Pisa, Italy; s.petruccelli@ao-pisa.toscana.it (S.P.); p.carrai@ao-pisa.toscana.it (P.C.); paolo.desimone@unipi.it (P.D.S.); 3 Programma Monitoraggio delle Malattie Parassitarie, AOU Pisana, 56124 Pisa, Italy

**Keywords:** *Toxoplasma gondii*, solid organ transplant, liver transplantation

## Abstract

*Toxoplasma gondii* (TG) is one of the most widespread intracellular parasites in the world, despite the slight declining trend in industrialized countries. Whilst the infection is often asymptomatic in immunocompetent hosts, in immunocompromised patients such as organ transplant recipients it can have important clinical sequels with even fatal consequences. We retrospectively reviewed 568 primary liver transplants (LT) from deceased donors from 2012 to 2017. Data were analyzed adjusting for year, gender, and age. The study objective was to assess the incidence of post-transplant TG infection and adherence to international guidelines for primary chemoprophylaxis. Prior to transplantation, 42.4% of recipients tested seronegative and 56.5% seropositive, while 36.6% of donors were seropositive and 40.4% showed undetermined serology. Anti-TG antibody titer was higher in patients born abroad (71.4%) versus Italy (54.8%). Among recipients at high risk of post-transplant TG infection, 82.7% of them received chemoprophylaxis, while in 17.3% of cases no prophylaxis was administered. At a mean (SD) follow-up of 21.2 (12.4) months no case of TG infection has been observed. Despite the low rate of adherence to recommendations, prophylaxis of high-risk LT recipients provides control of post-transplant TG infection risk. Review of current guidelines is warranted for low-risk populations.

## 1. Introduction

*Toxoplasma gondii* (TG) is an endocellular protozoan of the Apicomplexa phylum and one of the most common parasites infecting warm-blooded animals including humans [1,2,3,4]. Currently, its seroprevalence is decreasing in industrialized countries (USA and EU) and in younger individuals [1,2,3,4]. 

Whilst in immunocompetent individuals toxoplasmosis is often asymptomatic or limited to cervical lymphadenopathy or flu-like illness [5], it can be life threatening in immunocompromised patients and in those on immunosuppression [6,7,8,9,10]. TG is most typically transmitted from TG-seropositive donors (D+) to TG-seronegative patients (R−) (i.e., donor-recipient mismatch). However, reactivation of previous latent infections and primary infections have also been reported in transplant recipients [11,12,13,14,15]. To note, post-transplant reactivation may also occur when donor and recipients are both TG-seropositive [16,17]. 

In untreated seropositive recipients, reactivation of latent toxoplasmosis can be severe with fatal outcome [15,18]. For this reason, in allogeneic haematopoietic stem cell transplantation (HSCT) chemoprophylaxis is recommended in seropositive recipients prior to transplantation, whilst it is usually administered only to mismatched (D+⁄R−) recipients of solid organ transplants (SOT) [15].

The rate of infection is reported to be higher in heart and heart-lung transplants than in other SOT recipients [15,19,20,21,22], probably due to predilection of TG bradyzoites for muscle tissues and including myocardium [23].

Although clinically overt toxoplasmosis has been reported in a lower number of liver transplant (LT) recipients than in heart and kidney transplant patients, it can be fatal [24] with reactivation of latent infection being the leading cause of TG-related morbidity and mortality after LT [5,15]. 

In Europe, preventive measures include TG serology in donors and recipients prior to transplantation, in order to single out patients at risk of primary or reactivated infection [15,23]. Unlike France, where a national registry for toxoplasmosis was started in 1978 (French National Institute for Public Health Surveillance and National Reference Centre for Toxoplasmosis), in Italy, information about this infection is provided mainly by regional studies in pregnant women [25,26,27]. To note, serological screening of donors and recipients can be inconclusive since patients on immunosuppression fail to produce significant titers of specific antibodies [28]. In the absence of chemoprophylaxis, the incidence of toxoplasmosis in mismatched patients can be 50–70% [29,30]. Infection of seropositive patients from seropositive donors (D+/R+) is possible, but it may be hard to differentiate graft-related transmission versus reactivation of latent infection [31]. 

Diagnosis of toxoplasmosis in SOT is based on integration of clinical symptoms, radiology especially when the central nervous system (CNS) is involved, and serology, as well as on demonstration of parasites or DNA in blood, body fluids and tissues by polymerase chain reaction (PCR) [15,32]. On the opposite, diagnosis of a reactivated latent infection is based either on increased immunoglobulin (Ig) G titers with negative IgM and IgA, or on Western blot (WB) comparative analysis between pre- and post-transplant sera. High IgG avidity indicates TG reactivation rather than primary infection. In a survey by Fabiani et al. [33], the most frequent method for diagnosis of toxoplasmosis was indirect analysis (11%), although there was no indication about the method used in the vast majority of cases (77.8%) [33]. Serological and PCR follow-up is recommended in high-risk patients (D+/R− mismatch), while in other SOT categories and in HSCT recipients it should be limited to high-risk periods after transplantation due to its low risk-to-benefit ratio [15].

The aim of this study was to evaluate the impact of TG infection in LT patients admitted to the Transplantation Unit of Pisa University Hospital, Italy. Data were analyzed adjusting for year, gender, and age class. We also investigated adherence to international guidelines for TG prophylaxis after LT and reviewed the current recommendations about prevention of toxoplasmosis after LT. All patients are constantly followed-up at our center to detect post-transplant TG infection. 

## 2. Results

### 2.1. Patient Demographics

A total of 568 patients were included in the current analysis; 421 (74%) were male (mean age 53.6 ± 7.6) and 147 (26%) female (mean age 50.8 ± 10.3). Overall, mean (±SD) age of patients was 52.9 (±8.4) years (range 20–70). The vast majority of patients (504, 88.7%) were born in Italy, whilst 64 (11.3%) in other EU or non-EU countries. For foreign patients, most frequent countries of origin were Romania (22, 3.9%), Moldova (13, 2.3%), and Albania (9, 1.6%). Other countries were Morocco, China, Egypt, Liberia, Philippines, India, Bangladesh, Russia, Ukraine, Greece, Japan, Israel, France, Bosnia-Herzegovina, and Georgia. A total of 68 LT patients were assessed in 2012, 95 in 2013, 92 in 2014, 107 in 2015, 128 in 2016, and 78 in 2017.

Data on TG serology were grouped on the basis of donors’ immune status (positive, negative, or undetermined for anti TG antibodies), gender (male/female), and treatment adherence for at least three months after transplantation.

### 2.2. Prevalence of T. gondii Antibodies in Transplant Recipients According to Age and Serostatus

**Figure 1:**
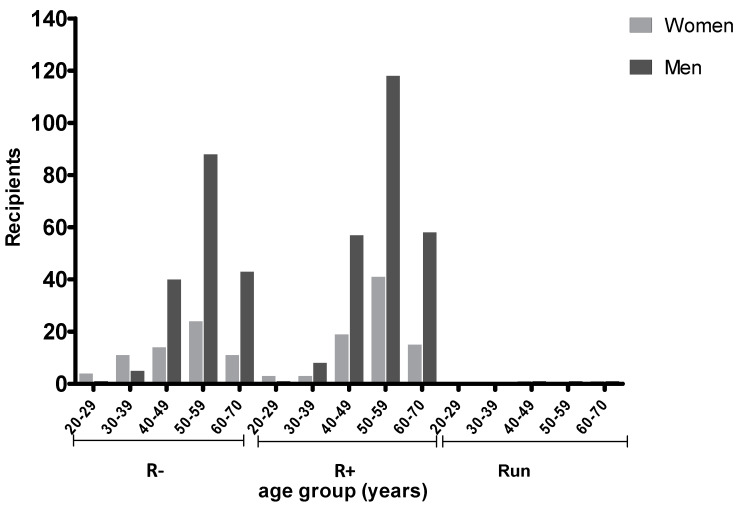
Recipient *Toxoplasma gondii* (TG) serostatus according to age and gender. R− (seronegative); R+ (seropositive); and Run (undetermined serology).

The pre-transplant recipient TG serology was stratified according to age and gender as shown in Figure 1. 

Most recipients were in the age range 40–70 (532, 93.6%). Namely, 271 (47.7%) were between 50 and 59 years, 132 (23.2%) between 40 and 49, and 128 (22.5%) between 60 and 70. Among the recipients, 241 (42.4%) were negative and 321 (56.5%) positive to anti-TG on initial screening. In 6 (1.1%), patient serology was undetermined. In seronegative recipients, mean (±SD) age was 53.7 (±9.0) for males and was significantly higher than for females (49 ± 11.5; *p* < 0.05). Women between 20 and 39 years showed lower IgG prevalence (28.6%) versus men (60.0%) of the same age group, but the difference was not significant (*p* = 0.059). No difference was observed for seropositive patients. Prior to transplantation, 111 of 271 (40.9%) recipients between 50 and 59 years were seronegative and 159 (58.7%) were seropositive; one patient was undetermined. In this age range, 118 out of 159 (74.2%) seropositive patients were male and 41 (25.8%) were female. 

**Figure 2:**
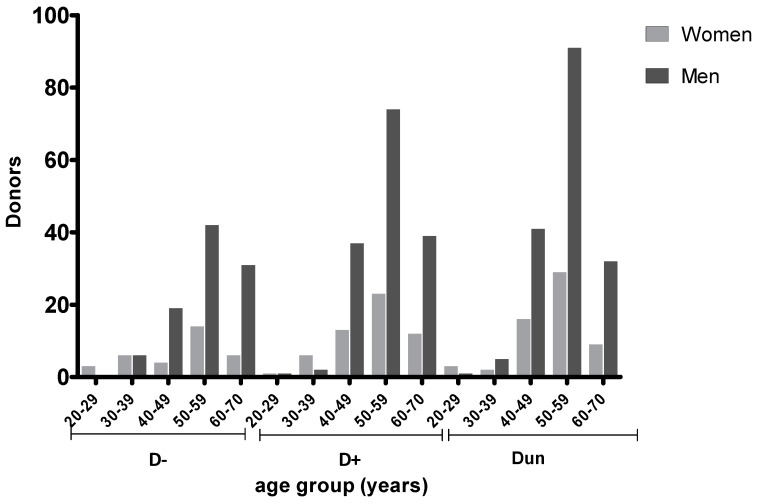
Donor TG serostatus according to age and gender. D− (seronegative); D+ (seropostive); and Dun (undetermined serology).

The majority of donors were in the age range 40–70 (533, 93.7%) with a higher prevalence in the 50–59 year group (273, 48.0%) (Figure 2). 

Anti-TG antibodies (IgG) were detected in 36.6% of donors. As shown in Figure 2, a high proportion (229, 40.2%) of donors had undetermined status, mainly (120, 21.1%) in the age range 50–59. In this group of patients, it was therefore not possible to estimate the actual prevalence of anti-TG antibodies. 

Overall, 159 (27.9%) recipients received primary chemoprophylaxis with trimethoprim- sulfamethoxazole (TMP/SMX) (Table 1), irrespective of donor serology.

Out of 156 recipients at risk of seroconversion, 129 (82.7%) received chemoprophylaxis with TMP/SMX according to guidelines. In 27 (17.3%) such patient prophylaxis was not administered.

In 132 cases (23.2%), the donor was seropositive and the recipient was seronegative (D+/R−). Most recipients in this group (122, 93.1%) received primary chemoprophylaxis. Ten of them (7.6%) were potentially at risk of seroconversion (as chemoprophylaxis was not administered).

In 18 cases (3.2%), a donor with an undetermined immunological serostatus was matched with a seronegative recipient (Dun/R−); chemoprophylaxis was administered in 5 out of 18 patients (27.8%). In four cases, donors and recipients with undetermined serostatus were matched. 

No patient developed acute toxoplasmosis during the follow-up period. Seventy-four donor-recipient matches (13.0%) were TG positive (D+/R+), and two of them were administered prophylaxis.

### 2.3. Toxoplasma gondii Prevalence in Native and Foreign LT Patients 

The seropositivity rate of the anti-TG antibodies in our study was significantly higher among recipients born outside Italy (71.4%) than among Italian patients (54.8%) (*p* = 0.017). In the former group, the positivity rate resulted higher in female (80.0%) than in male (65.8%, *p* = 0.35) patients. A significant difference was observed between Italian and foreign seropositive females (OR = 3.80; *p* = 0.012). Recipient TG serostatus according to ethnicity and gender is shown in Table 2.

Among Italian patients, two (1.7%) females and five (1.3%) males had undetermined serology. No undetermined serology was detected among foreign recipients.

## 3. Discussion

Toxoplasmosis following LT is uncommon. During a period of 40 years, only 31 cases have been reported, and of these only 10 were the consequence of donor-to-recipient mismatch [24]. As a result, prophylaxis is sporadically prescribed by transplant physicians, despite life-threatening cases of post-transplant toxoplasmosis having been described [24].

According to Fernandez–Sabe et al. [20], LT recipients have a low incidence of post-transplant toxoplasmosis. In a cohort of 4872 recipients followed for nine years (2000–2009), only four (0.08%) developed toxoplasmosis, suggesting that this infection is relatively uncommon. A systematic review of the international literature was recently performed and reported 20 cases of primary infection or reactivation in 17 publications. Despite the fact that treatment was administered in 70% of cases, nine patients died after the occurrence of signs of toxoplasmosis [34].

Experimental studies have shown the presence of *Toxoplasma gondii* cysts in the liver of chronically infected mice [24]. Based on this observation, pre-transplant serological screening of liver donors and recipients is justified, and chemoprophylaxis of D+/R− recipients is to be encouraged, which is not uniformly implemented in Europe [23]. Due to immunosuppression, TG can reactivate in transplant patients causing serious consequences with high morbidity and mortality. Toxoplasmosis frequently occurs within three months after transplantation, even though it has occasionally been reported as early as two weeks after surgery [15]. 

In our study, males represented 74% of the LT patients, most of them in the 50–59 year age class. Some studies focused on gender difference in SOT recipients [35,36,37,38]. Our results are in agreement with Puoti et al. [39], who in a meta-analysis of 43,320 transplants performed in Italy in the period 2002–2015 observed that recipients of organs are mainly males, probably reflecting a gender bias in the incidence of transplant-related pathologies. It should be interesting to analyze whether gender may somehow influence survival results of transplant grafts and patients in a larger sample group. 

In the present study the prevalence of an anti-TG antibody positive status was 36.6% for donors and 42.3% for recipients, with a high proportion of undetermined samples among donors (40.3%). Several reasons may account for this latter finding, such as the fact that organ procurement takes place at local hospitals where anti-TG testing is not always available 24/h, and due to multiple blood transfusions before organ procurement. Conversely, an undetermined serology was very low (1.0%) in transplant recipients whose pre-operative work-up is routinely performed.

The prevalence of anti-TG antibodies was higher in our recipient sample as compared to the general population living in our regional area and whose overall prevalence was 28.2% [40]. This is probably due to the proportion of foreign patients included in the current study. In a meta-analysis on parasitic infections occurring after SOT, LT represented 11.7% of all toxoplasmosis cases reported in the literature over a 20-year period, being less frequent than in kidney transplantation which contributed for over 46% of all cases [33]. 

According to a survey carried out in several EU countries, serologic screening of solid organ donors (heart, kidney, or liver) was performed in all countries, but was considered mandatory only in seven countries (France, Greece, Italy, Romania, Slovakia, Switzerland, and Turkey) [23]. In most centers (24/26), SOT recipients were routinely screened for toxoplasmosis [23]. The Italian Association for the Study of Liver Disease (AISF) recommended TG serology as a second level screening to be performed in all patients eligible to LT [41]. 

In terms of adherence to guidelines, primary prophylaxis was administered in most recipients at risk of seroconversion. A low proportion (1.8%) of them, and corresponding to 7.6% of D+/R− mismatches, did not receive chemoprophylaxis. In spite of this, seroconversion or reactivation have not been observed in our population during further follow-up. This might be the result of daily habits (e.g., hand washing and use of cooked meat), but more likely of reduced immunosuppressive regimens in recent eras [42].

As regards the serological status of recipients, it is not surprising that foreign recipients showed higher positive rates versus Italian patients, since in our country, as well as in other western countries, TG seroprevalence is declining [4]. 

In a study carried out in Turkey, 67.5% of 40 transplant recipients resulted seropositive for TG and were therefore at risk of reactivated infection. However, over a period of four months only three patients tested PCR positive, corresponding to 11.1% of those at risk and suggesting reactivation [43]. The authors underlined that anti-TG prophylaxis had been withdrawn prior to testing in all of the PCR-positive patients, whereas after treatment administration PCR reverted to negative and the symptoms resolved. In the D+/R− group PCR resulted negative, ruling out primary infection [43]. 

Universal prophylaxis with TMP/SMX is considered useful to remedy the need for TG serology [44]. 

According to Derouin et al. [15] (2008), in cases of D+/R− mismatch or D+/R+ SOT prophylaxis is recommended for one year with TMP-SMX or Atovaquone, including LT. In the AISF consensus paper on management and treatment of infections after LT, use of TMP-SMX to prevent *Pneumocystis jirovecii* infection is encouraged because most *Toxoplasma gondii* and *Nocardia* spp infections can be prevented as well [41]. However, prophylaxis does not eliminate the risk of toxoplasmosis. A case of chorioretinitis has been reported despite TMP/SMX prophylaxis in a D+/R− mismatched LT [45]. Although the symptoms presented four months after discontinuing prophylaxis, the absence of tachyzoite-specific antibody and the lack of evidence on other transmission pathways suggested that this patient had acquired infection through the liver graft [45]. Interestingly, in a recent review focusing on treatment of toxoplasmosis the authors did not mention use of any post-transplant chemoprophylaxis [46].

Although multicenter validation of our results would be strongly needed, the current study captures real life clinical practice and might contribute to critical appraisal of existing guidelines for management of toxoplasmosis after LT. Due to type of graft and lower immunosuppressive regimens, LT recipients are not comparable to other SOT patients and might need a tailored approach.

## 4. Materials and Methods

### 4.1. Study Population

The study consisted of retrospective review of all adult, primary LT patients who underwent LT between 2012 and 2017 (collected informations consisted of demographic data such as age, gender, ethnicity, chemioprophylaxis, and laboratory results of anti-*Toxoplasma* blood test when available). Data were anonymized and the study was approved by the Local Ethics Committee of Pisa University Hospital (former Comitato di Bioetica dell’Azienda Ospedaliero Universitaria Pisana), approval number 3316 released on 23 June, 2011.

Patients who died of TG-unrelated post-operative complications were excluded from the analysis, as well as patients who were re-transplanted during the observation period and those with incomplete medical information (n = 11). 

Post-transplantation chemoprophylaxis was administered to 159 out of 409 recipients (Table 1), and consisted of TMP/SMX for three months at the dose of 400 mg/day in a single dose, in agreement with international guidelines [34]. 

### 4.2. Patient Serological Testing

Serological tests were performed to assess the anti-TG status of both donors and recipients at transplantation. For donors, the serological analyses were performed at procurement hospitals. When serology was not available, the donor serostatus was indicated as undetermined.

Post-transplantation, all LT recipients underwent routine serial monitoring of anti-TG IgG and IgM at months 3, 6, 12 and yearly thereafter. For IgM, a commercially enzyme linked fluorescent immunoassay (ELFA), the VIDAS TOXO IgM kit (bioMérieux, Marcy-l’Etoile, France) was used. The level of IgM was expressed as sample Index and cut-off values were as follow: Index < 0.55 negative, 0.55 ≤ Index < 0.65 undetermined, and Index ≥ 0.65 positive. Equivocal samples were re-tested only in case of clinical suspicion. The VIDAS TOXO IgG II kit (bioMérieux, Marcy-l’Etoile, France) was used to measure the level of anti-TG IgG in plasma by the ELFA assay. Samples were considered negative when the value was <4 UI/mL, undeterminate for values ≥ 4 to <8, and positive when ≥8. IgG positive samples were analyzed for avidity test (VIDAS TOXO IgG Avidity, bioMérieux, Marcy-l’Etoile, France). Cut-off values were as follow: Index < 0.200 IgG weak avidity; 0.200 ≤ Index < 0.300 intermediate avidity; and Index ≥ 0.300 IgG high avidity.

### 4.3. Statistical Analysis

Categorical variables were analyzed using the χ^2^ test or Fisher’s exact test as appropriate. Quantitative data were compared across two groups using the Mann–Whitney’s test (or ANOVA and Tukey’s multiple comparison test). A logistic regression method was used to estimate odds ratios (OR) and 95% confidence intervals (CI) in order to measure the association between positivity to anti-TG and gender within ethnicities. A *p* value of <0.05 was considered statistically significant. Analyses were conducted with GraphPad Prism v.5.0 (La Jolla, CA 92037, USA).

## 5. Conclusions

In conclusion, despite the low incidence of TG infection after LT, the results of the present study underline the need to screen both donors and recipients with the aim to implement prophylaxis mainly for mismatched cases or recipients at risk of reactivation. Use of chemoprophylaxis in low-risk recipients requires updating of current recommendations, especially in view of the lower immunosuppressive schedules implemented in clinical practice. 

## Figures and Tables

**Table 1:** Transplant donor and recipient groups according to serological status and administration of primary chemoprophylaxis.

Group	LT with Prophylaxis	LT w.o. Prophylaxis
D−/R−	24	67
D−/R+	1	39
D+/R−	122	10
D+/R+	2	72
D+/Run	1	1
Dun/R−	5	13
Dun/R+	3	204
Dun/Run	1	3
Total	159	409

+, seropositive; −, seronegative; and un, undetermined serology.

**Table 2:** Recipient TG serostatus according to ethnicity and gender.

Patient Ethnicity and Gender	Italian N. (%)	Foreign N. (%)	Adjusted OR* (CI)	*p*-Value
Female R−	58 (47.9)	5 (20.0)		
Female R+	61 (50.4)	20 (80.0)	3.80 (1.34–10.80)	0.012
Male R−	163 (42.4)	13 (34.2)		
Male R+	216 (56.3)	25 (65.8)	1.45 (0.72–2.92)	0.297
Total	498	63		

+, seropositive; −, seronegative; and un, undetermined serology.

## Abbreviations

AISF	Italian Association for the study of liver disease
CI	confidence intervals
CNS	central nervous system
HSCT	allogeneic hematopoietic stem cell transplantation
Ig	Immunoglobulin
LT	liver transplants
OR	odds ratios
PCR	polymerase chain reaction
SOT	solid-organ transplant
TE	toxoplasmic encephalitis
TG	*Toxoplasma gondii*
TMP/SMX	trimethoprim/sulfamethoxazole
WB	Western blot
